# Imperfect Development a Factor in Genesis of Diseases of Women

**Published:** 1913-07

**Authors:** B. S. Moore

**Affiliations:** Atlanta, Ga.


### IMPERFECT DEVELOPMENT A FACTOR IN GENESIS OF DISEASES OF WOMEN.

By B. S. Moore, M. D., Atlanta, Ga.

We have had in the past few years many, and most excellent papers on most every subject in medicine and surgery, and especial attention has been given preventive medicine.

In this great field of scientific work there is one subject which I think has been neglected to a great extent, and that is the influence of imperfect development as a factor in genesis of diseases in women.

Eliminating infection and bad management during labor, imperfect development of the generative organs of women is the cause of all of the diseases of those organs, either directly or indirectly. I may add here that it is the cause also, of most of the complications during and after labor.

We have had many diseases of women classified and successful treatment for most of them worked out along scientific lines. The fact remains that it is all repair work, and in most cases, it is a removal of some part. I am sorry to say that very, very often parts, especially one or both ovaries are removed, when from a standpoint of justice to the patient, it amounts to criminal work. I do not mean to go into the many diseased conditions we find of those parts of the female, for so much has been written along these lines, but I want to appeal to the profession at large and the general practitioner in particular, to if possible stimulate, at least, a few to take more interest in advising the parents of the importance of giving young girls more attention, especially physical care of their girls during the proper years of their development.

I will not take up the comparative anatomy of man to that of other animals or even a comparison of races, but let us take a comparative view of the different classes of the same race of people.

We will take the two clases: Those who earn a living by the use of their brains, or have it already earned for them by their fathers. The other class—those who have to earn a living by manual labor. Mention here that this applies to women who actually work, not part of that class.

Now among the class of people who can afford to give their children every attention, there are fewer children born than among the less fortunate classes, and in the more fortunate classes many more children live to the age of puberty, for with the less fortunate class the law of the "Survival of the fittest" is not so much interfered with. Therefore, we find relatively more uterine diseases among the children of the rich.

Ashton says—"The lower classes receive less skillful attention during and after confinement, consequently septic infection is comparatively frequent and traumatism more often found in the lower classes and is either improperly repaired or neglected altogether. The higher classes on the other hand, suffer more from neurasthenial conditions and various reflex symptoms, which are more or less dependent upon their environment and habits of life. Furthermore, women of the lower classes are less affected by the diseases from which they suffer and it is not uncommon to find them attending to the duties and labors which are consequent to bringing up large families, while suffering from local conditions which would make an invalid of a woman in the higher walks of life. Finally, certain occupations are likely to result in pelvic disease, and we find that women who work in factories and stores, where they are required to stand continuously for hours at a time, frequently suffer from uterine displacements."

I do not agree that we find more traumatism in the lower classes, relatively speaking. Take the same number of women out of each class and the same number of children born to each and regardless of skill and care given to the higher classes, you will find a better and healthier condition of the pelvic organs of the lower classes, for they are better developed to start with.

Certainly occupation has its influence upon the pelvic organs of women, such as work in stores and factories, where the women have to stand for hours, but the ill effects are due to the fact that the general health is impaired and the pelvic organs are indirectly affected. Of course, this is worse with girls during the years of adolescence and in women who have lacerated perineum and subinvoluted conditions of the uterus following labor.

Education by our modern system has a most injurious effect upon the general and sexual strength of women, inasmuch as the system is not properly balanced as to mind and body. Too little attention is given to the development of the general and sexual health to give young girls a polished education. Enough attention is not given by the parents or the educator to regulate the amount or character of work to suit the health and temperament of the individual and no consideration is given to the necessity for special care at the time of puberty and years of adolescence. Young girls are sent to school or college and subjected daily to long hours of study in badly ventilated rooms, regardless of their age or physical condition or the demands of their sexual development.

"In one word, it is to the cramming and high pressure system of education, together with its environment, that I attribute much of the menstrual derangements, sterility and barrenness of women, the absence of sexual feeling, the aversion to maternity, the too often lingering convalescence from a first labor, which is frequently the only one, and the very common inability to suckle their offspring. From this cause came most of my unmarried patients, with nerve prostration, with their protean mimicry of uterine symptoms, unmarried very often because they are not well enough to wed. If woman is to be thus stunted and deformed to meet the ambitious intellecual demands of the day, if her health must be sacrificed upon the altar of her education, the time may come, when to renew the wornout stock of this Republic, it will be needful for our young men to make matrimonial excursions into lands where educational theories are unknown."—(Goodell.)

There is very little doubt but that here in the United States this practice is more marked than in Europe and, no doubt, it is practiced here in the South more than any other part of the United States. For, in my opinion, it is one of, if not the most, vital cause of many of the conditions of the pelvic organs of women, known as the Diseases of Women.

The generative organs are not essential to the life of the individual, but they are the last to develop, and they lie practically dormant from early childhood until the girl reaches the age of puberty. Then during the years of adolescence, from 10 to 16 years, they develop and make a large demand upon the entire system.

We know that our domestic animals will not stand any continuous strain at even ordinary work, during or even before development and the consequence is that they are not worked at that age.

Now the same thing applies to the human body and surely we should give our young the consideration that is given to the domestic animals of the land.

To be sure of full development, a girl must have a surplus of vitality during the period of development. If this surplus of vitality is not present, or has been used, the generative organs will fail to develop sufficiently to perform their functions normally. If a girl is pushed at school or her energy used up by constant contact with older and more highly educated people so as to keep her on a mental strain to keep the pace, she is apt to use up her surplus.

If this train, either mental or physical takes place from the age of ten to sixteen, the girl is almost certain to have the following symptoms manifest themselves: Leucorrheal discharge and irregular and painful menstruation and upon local examination we find often an undeveloped condition of all the parts, clitoris, small, undeveloped and buried under an adherent labia minora. This, in itself, causes many reflex disturbances. I have seen such a condition cause epileptic fits; a small undeveloped cervix, a flexed and often infantile uterus. The leucorrheal discharge comes from an already more or less chronic endocervicitis.

I might mention many other undeveloped conditions you find in these cases. A severe illness, bad hygienic surroundings or anything that will bring about an enemic condition during the years of development, is very apt to result in imperfect development of the generative organs. Here I would like to mention or bring to the notice of the profession a condition which I fear has been looked upon too lightly. It is the havoc wrought by the acute infectious diseases in the female during the years of development and adolescence. You often have a patient come to you the picture of physical beauty and development. She complains of scanty, irregular or painful menstruation. Her menstrual history from the beginning is normal for first two or three years. That is, first evidence was stain on clothes when 13 years of age. Flow lasts about five days, comes about every 28 days, no pain. At age of 16 years had a slight attack of measles. Since that time menstruation has been very scanty, irregular and painful. On examination you find the cervix small, undeveloped, the same with the uterus and its appendages, if the patient is one in whom you can outline the tubes and ovaries by digital examination.

I am sure if we all appreciated this arrest of development of the generative organs in women by the acute infectious diseases, we would insist on a better quarantine system in all of our communities. I am sure the strictest form of guarantine in our cities or any locality where one of the diseases prevailed would be a cheap price for the harm done in this way as well as the death toll usually taken.

A young woman may appear to be in good health and seem to be well developed, and yet she may be imperfectly developed so far as her generative organs are concerned, and will come to you with a history of slight leucorrhea, she may also complain of painful menstruation, growing worse each month. They may sometimes come with only a history of a dragging feeling about the pelvis or a pain in the left side, usually with extreme constipation and often fissue in ano or hemorrhoids, and on examination a well marked case of imperfect development will be found, with a chronic endocervicitis, the usual dysmenorrhea being absent, or not yet developed.

At times the only indications of imperfect development and disease of the glands of the cervix is a pain in the left side and an uncontrollable nervousness, with more or less depression and hysteria, as the woman grows older. If the case is not treated the menstruations soon become irregular and profuse, the latter symptoms being due to the constant irritation resulting in congestion and abnormal vascularity and finally to the development of fungus or polypoid growths in the endometrium above the os internum. Typical cases of imperfect development are to be found, especially among the graduates of our female colleges, for to graduate from such schools, large demands are made on the general physique, and unfortunately for the students the hardest part of this work is done during the years of adolescence. I feel that the best rule that could possibly be added to those governing the pupils of female colleges, is that every applicant should be fully developed physically. The abnormal changes gradually produced by cervicitis and the more rare condition endometritis will very often result in sterility.

Suppose pregnancy should occur in an imperfectly developed uterus wher the os interi is not only imperfectly developed but rendered hard and incapable of dilating sufficiently to allow the head of the child to pass without lacerating the cervix. The tissue being filled with more or less diseased glands and follicles, fails on this account to heal and the result is that the existing disease is aggravated and the resisting ability of the parts affected is reduced, involution is delayed, the uterus remains soft and heavy, the ligaments remain relaxed or have lost their tone—these conditions cause many of the cases of distortion of the uterus called displacements, etc. Besides the existing catarrhal disease, this condition is most inviting to sepsis.

"Excluding new growths, sepsis, unclean examination and application just before, during, and after labor and abortions, this condition of imperfect development is the real cause of most of the local conditions from which women suffer." (G. Wylie.) The condition may affect the ovaries and cause imperfect and abnormal ovulation at times resulting in Duemoid cepts, in sterility or reflex nervous disturbances.

This weakened condition of the glands and underlying tissues is a most favorable condition to the growths known as new growths, such as fibromata, cancer, etc.

Now, if I am not misled by my own and the observations of others, much can be done, which is not at present, toward the prevention of diseases of women. The great importance of keeping children in good health has been dealt with most ably but I think too much stress has been laid upon physical rest, especially in bed, during the menstrual period. The very important fact that during the period of development between the ages of ten and sixteen, or better eighteen, a girl must not only have good health and strength, but this health and strength must not be used up in mental or social development. If it is, the generative organs are most certain to suffer and fail to develop.

Rest during menstruation may relieve the pain, but if it shuts the girl up and deprives her of moderate out-of-door exercise, it does more harm than good, often being the cause of bringing about abnormal conditions and harmful habits, which would not have happened had she been playing out-of-doors, occupied mentally by innocent, playful thoughts.

The very common idea that most of the diseases of women come from displacement, falls, etc., should be replaced by a broader view. The main points to bear in mind in the care of young girls are:

FIRST. Keep the general health good, the bowels regular during the period of active development; let healthful out-of-door play and pleasing mental occupation take the place of an indoor life, forced cramming and stimulating contact with older people, recognizing the fact that a girl has brains to be developed and that they can best be developed after her generative organs have been developed, that to develop their brains and have them so-called accomplished at the expense of their generative organs is to given them the worst possible preparation for a happy and useful life.

The importance of recognizing the influence of imperfect development or the failure to recognize it, is a great handicap to treatment of diseases of women, i. e., you treat a case of dysmenorrhea and sterility as being caused by flexion, when you straighten the uterus your work is done. But suppose you look upon the small uterus with its semi-raw os and contracted canal as being due to imperfect development, our aim would be to cure the local disease of the glands and follicles and then stimulate the development of the uterus, and you will recognize, unless you keep the patient's general health good, and in other ways stimulate healthy development, it will only be a matter of a short time before the abnormal condition will return.

I do not for one moment make the claim, that if women were perfectly developed, especially their generative organs, that all their physical ailments would be cured for all time. However, a very large percentage of abnormal conditions now found in the generative organs of women would be cured and now where domestic happiness is impossible, health and happiness would reign supreme.

In the human race, as man develops mentally, the ratio of birth diminishes and so does the physical development of those who are born. The conditions are due undoubtedly to unbalanced development of mind and body, there are of course many exceptions to this rule.

The foregoing statement is so manifestly true that we need not spend hours looking up statistics to prove the truth thereof, for it is a common saying: "Oh! so and so is too smart to have children." I do not agree with many as to the origin of this saying, if the opposite view from the one I take were true, I am sure the ratio of births would be reversed as to class. I believe in most cases where there are none, or only one child, in the family of highly intellectual people, it is due to physical inability rather than to the willful intent of man.

I am sure that there is a degree of satisfaction in the life of every man and woman, which can be attained only by being a party to the creation of a living being who is at least in part made after their own image, which we are taught to believe is made in the image of God.

